# Propolis Controlled Delivery Systems for Oral Therapeutics in Dental Medicine: A Systematic Review

**DOI:** 10.3390/dj11070162

**Published:** 2023-06-29

**Authors:** Andressa da Silva Barboza, Juliana Silva Ribeiro de Andrade, Monika Lamas Ferreira, Carla Lucía David Peña, Juliê Silveira da Costa, André Ricardo Fajardo, Rafael Guerra Lund

**Affiliations:** 1 Graduate Program in Dentistry, Pelotas Dental School, Federal University of Pelotas, Gonçalves Chaves Street, 457/Rm 702-3, Downtown Pelotas, Pelotas 96015-560, RS, Brazil; andressa.barboza@ufpel.edu.br (A.d.S.B.); juliana.r.andrade@ufsc.br (J.S.R.d.A.); monika.ferreira@ufpel.edu.br (M.L.F.); carla.pena@ufpel.br (C.L.D.P.); 2 Department of Dentistry, Federal University of Santa Catarina (UFSC), Av. Delfino Conti, s/n-Trindade, Florianópolis 88040-900, SC, Brazil; 3 Laboratory of Technology and Development of Composites and Polymeric Materials (LaCoPol), Center for Chemical, Pharmaceutical and Food Sciences, Federal University of Pelotas, UFPel, Campus Capão do Leão, Pelotas 96010-900, RS, Brazil; julie.costa@ufpel.edu.br (J.S.d.C.); andre.fajardo@ufpel.edu.br (A.R.F.)

**Keywords:** propolis, oral bone regeneration, drug delivery, guided tissue regeneration, tissue engineering, systematic review

## Abstract

This systematic review synthesizes the existing evidence in the literature regarding the association of propolis with controlled delivery systems (DDSs) and its potential therapeutic action in dental medicine. Two independent reviewers performed a literature search up to 1 June 2023 in five databases: PubMed/Medline, Web of Science, Cochrane Library, Scopus, and Embase, to identify the eligible studies. Clinical, in situ, and in vitro studies that investigated the incorporation of propolis as the main agent in DDSs for dental medicine were included in this study. Review articles, clinical cases, theses, dissertations, conference abstracts, and studies that had no application in dentistry were excluded. A total of 2019 records were initially identified. After carefully examining 21 full-text articles, 12 in vitro studies, 4 clinical, 1 animal model, and 3 in vivo and in vitro studies were included (n = 21). Relevant data were extracted from the included studies and analyzed qualitatively. The use of propolis has been reported in cariology, endodontics, periodontics, stomatology, and dental implants. Propolis has shown non-cytotoxic, osteoinductive, antimicrobial, and anti-inflammatory properties. Moreover, propolis can be released from DDS for prolonged periods, presenting biocompatibility, safety, and potential advantage for applications in dental medicine.

## 1. Introduction

Odontogenic infections, such as dental caries, periodontal diseases, endodontic infections, and dental abscesses are very common problems in dental medicine, with caries as the most common infectious dental disease in the world. They mainly involve an interaction between the microbial biofilm and tooth structure or oral tissues. When not properly treated, these infections can promote inflammation and consequently irreversible damage to oral tissues [1]. In general, conventional treatments involve removing the affected tissue and replacing it with filling materials and surgical approaches [2]. Despite these treatments being effective, they may not be ideal as they do not biologically replace the lost tissue. So, the development of new alternative methods aiming to provide antimicrobial, anti-inflammatory, and biological properties during the treatment of oral lesions are considered promising in dental research [2]. However, these methods also include systemic or local administration of high doses of drugs leading to antibiotic resistance, patient sensitivity, and possible side effects. In this context of aiming to reduce these drawbacks, the field of drug delivery systems (DDSs) has grown considerably in the last decades [3].

DDSs are an approach where an optimal amount of a drug is encapsulated, delivered, and acts on the exact site of the lesion [4]. They are formulated by combining drugs with biomaterials of lipidic, inorganic, and/or polymeric nature to provide a more effective way of delivering the compounds to targeted sites [5]. Their efficiency and safety are enhanced by controlling the rate, time, and location of drug release in the organism [4,6,7]. The development of DDSs has many advantages, such as improved solubility and bioavailability, increased pharmacological activity, stability, reduced toxicity, improved tissue macrophage distribution, sustained delivery, and protection from physical damage and chemical degradation [7]. In addition, the use of phytotherapy in DDSs is of great interest due to its low cost, availability, low or no adverse effects, and complex composition in bioavailable nutrients can be used not only for synthetic drugs but also for herbal medicines [8].

In this sense, propolis is a natural product produced by bees from trees [9,10]. It is a non-toxic natural resin with numerous pharmacological properties, including antimicrobial, anti-inflammatory, anticancer, antioxidant, and improved immune system effects [11,12,13]. These properties are mediated by a variety of bioactive compounds present in propolis, that act in different ways, therefore it possesses a complex mechanism of action. Its chemical composition may vary depending on the geographic location and the plants from which it was collected, but it generally includes a mixture of resins, waxes, essential oils, polyphenols, flavonoids, and phenolic acids, among other compounds [9,10,12].

Several studies report the use of this natural substance in most various dental formulations, such as oral and intracanal preparations, lip creams, membranes, and nanomaterials, and the results have proven its pharmacological activities [13,14,15,16,17,18,19,20,21,22,23,24,25]. Furthermore, according to previous studies, propolis may induce the repair and regeneration of bone [26,27], cartilaginous [8,28], and pulp tissue [12,29]. Based on these aspects, this research aims to systematically review the literature to obtain an updated overview of the use and effectiveness of DDSs containing propolis for oral applications.

## 2. Materials and Methods

### 2.1. Protocol and Registration

This systematic review was carried out following PRISMA statement guidelines [30]. It was registered on the Open Science Framework platform under registration DOI 10.17605/OSF.IO/V9FXT.

### 2.2. Research Question (PICO)

The research question (PICO) was “Is there an influence on the incorporation of propolis in drug delivery systems comparable to drug delivery systems with other substances or without propolis?”, where the following items where observed:

- P: the drug delivery systems DDSs
- I: application and efficacy of propolis in drug delivery systems, in dental medicine
- C: comparison between DDSs without propolis extracts and/or DDSs with other substances
- O: effectiveness of propolis-based DDSs for dental medicine
- S: clinical, in vitro and in vivo studies.

### 2.3. Eligibility Criteria and Selection Process

Investigations using propolis in DDSs or combined with other biomaterials, molecules, or stem cells in the dental medicine field were selected. The inclusion criteria were papers evaluating propolis-based DDSs for biomaterials. The following items were considered as exclusion criteria: literature reviews, clinical cases, case reports, dissertations, thesis, conference abstracts, and studies that evaluated the actions of propolis-based DDSs in areas other than dentistry.

### 2.4. Information Sources and Search Strategy

The search was conducted in five electronic databases: Medline (PubMed), Web of Science, Scopus, Embase, and Cochrane. The final search was carried out on 1 June 2023 without any restriction of time or language. The search strategy used was appropriately adapted for each database and is listed in Table 1. The titles of all identified studies were screened by two independent reviewers, blind to each other (A.B. and J.R.) using the online system Rayyan QCRI (Hamad Bin Khalifa University, Doha, Qatar).

### 2.5. Data Collection Process

Abstracts were carefully appraised; studies that met the inclusion criteria or had insufficient data available in the title or abstract were selected for a full-text analysis. Disagreements reported on the eligibility of the included articles were resolved by consensus and by a third reviewer (M.F.). Reference lists of all the included studies were also hand-searched for additional studies.

### 2.6. Data Items

The study information, such as demographic information, enrollment criteria, study design, aims of the study, application in dentistry, type of biomaterials, type of propolis, characterization and origin, toxicity assessment, drug release, main results, presence of controls, and sample size were extracted by the reviewers (A.B. and M.F.).

### 2.7. Study Risk of Bias Assessment and Synthesis of Results

The risk of bias of the included studies was analyzed according to the RoBDEMAT tool [31] for laboratorial analysis. The clinical studies were analyzed according to Robbins-I [32] to non-randomized trials and Rob 2 to randomized clinical trials [33]. In addition, a qualitative synthesis of results was performed based on individual studies and is presented in the next section.

## 3. Results

### 3.1. Study Selection

**Figure 1:**
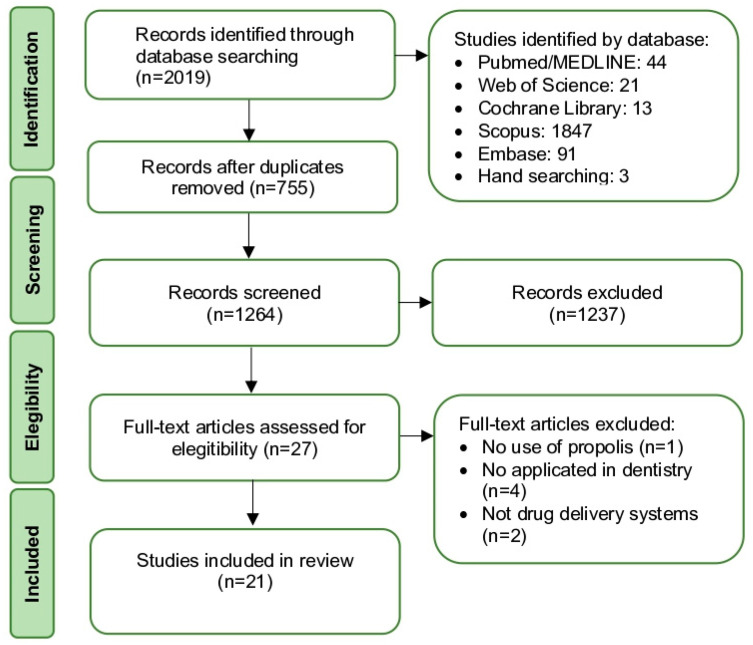
Flow diagram of the systematic review.

A flowchart illustrating this review’s search and selection is presented in Figure 1. The search resulted in the retrieval of 2019 articles. After the database screening and removal of duplicates, 1264 studies were identified. Then, 26 titles were screened, a careful examination of the full texts was performed and assessed to check if they were eligible for this systematic review. As a result, four studies were excluded because they did not fit the inclusion criteria and 21 studies were selected.

### 3.2. Study Characteristics

The characteristics of the included studies are described in Table 2. The studies were published between 2007 and 2021. Brazil was the country with the highest number of studies on propolis-based DDSs in dental medicine. Fourteen studies were conducted in vitro, followed by four randomized clinical trials, clinical and two in vitro studies, and only one evaluated under in vitro and in vivo (animal model) conditions.

Regarding application in dentistry, six studies had application in periodontics (periodontal pockets and guided tissue regeneration), followed by six in oral medicine (oral lesions), four in endodontics (pulp protection), two in cariology (anti-cariogenic agent), one in implantodontics, one in regenerative dentistry (hard tissue), and one in control oral infection. Eleven studies used the ethanolic extract of propolis, two used aqueous extract (2), one used ethyl alcoholic, and one used hydroalcoholic solution (1). All studies reported the origin of the propolis used, except three studies [18,34,35].

### 3.3. Results of Individual Studies and Results of Syntheses

Of the twenty-one selected studies, only three studies evaluated the toxicity of the materials. All studies had a control group during the tests pmed. Chlorhexidine was the most commonly found control substance (three studies). Only one study did not report the sample size [19]. The animal model study did not report the sample size calculation, despite reporting that they followed international protocol for studies in animal models.

Concerning drug release, propolis can be released from systems for long periods up to 7 days [20,29,36]. The main results reported the use of propolis in infected periodontal pockets, as it results in the production of higher quality secondary dentin with a lower inflammatory response [29], revealing a role in tissue regeneration as in the study by Simu et al. (2018) [8] which demonstrated an essential osteoinductive effect for mineralized tissue repair.

In studies involving periodontal [20,21,22] and endodontic [9,14,29] diseases, formulations containing propolis indicate a potentially beneficial anti-inflammatory and antimicrobial effect. Some studies show the antimicrobial activity of propolis against Gram-positive [17,18] and Gram-negative bacteria [21], especially Streptococcus mutans [15,18,23], in addition to its antifungal potential against Candida albicans [19]. Some bacteria, such as Streptococcus pyogenes and S. mutans, showed greater susceptibility to propolis compared to metronidazole [24]. Other studies [15,23] have evaluated the ability to prevent cariogenic biofilm compared to gold standard antibacterial agent chlorhexidine and antifungal (nystatin). Further to this, Borges et al. (2015) [25] reported that its incorporation increased the strength of the film matrix and mucoadhesiveness. In addition, it was reported that propolis reduced the inflammatory response and showed no side effects [11,35] which may have promising results especially in the field of stomatology, such as in the treatment of aphthous ulcers and lichen planus [35,36,37,38,39].

### 3.4. Risk of Bias in Studies

In vitro studies exhibited a high risk of bias concerning sample randomization, evaluation blinding, and sample size calculation (Table 3). However, a low risk of bias was observed in terms of presence of control group, statistical analysis, outcome reporting, and analysis standardization between groups. Non-randomized clinical study demonstrated a low risk of general bias (Table 4). For the randomized clinical trials (RCTs), a low risk of bias related to randomization [34,35,37], selection of reported outcomes, and measurement of outcomes was reported (Table 5).

## 4. Discussion

Numerous drug delivery systems (DDSs) have been developed for the local treatment and prevention of several diseases in the oral cavity [3,42]. These systems are a safe option as they drastically reduce the adverse reactions due to low doses administered directly at the site of action [11]. In addition, DDSs increase stability and solubility, which is interesting for the use of natural extracts [43]. Among natural compounds, propolis is an advantageous alternative to be used in DDSs due to its biodegradable nature, high tissue compatibility, and long-term release [29]. Therefore, the present study presents scientific evidence for the incorporation of propolis in controlled delivery systems as a therapeutic agent in dental medicine.

Propolis is widely recognized for its antimicrobial and anti-inflammatory properties. Although few studies were found using propolis in controlled drug systems in dentistry, it is possible to observe the diversity of areas in dentistry in which propolis can be applied for therapeutic purposes. Propolis composition can vary according to geographic and environmental conditions in which it is collected, as well as the solvents and parameters used during its extraction [23,25]. Therefore, spectrophotometric methods are important to characterize and standardize compounds present in propolis [29].

Propolis has three main compounds: flavonoids, cinnamic acid derivatives and terpenoids. The cinnamic acid derivatives, also known as phenolic compounds of propolis, include caffeic acid, rutin, quercetin, apigenin, chrysin, ferulic acid, cinnamic acid and galangin [29]. Flavonoids are a very important class of polyphenols, as they are plant compounds that have antimicrobial, antioxidant and anti-inflammatory properties [24]. Their anti-inflammatory property stimulates phagocytic activity and cellular immunity. Propolis contains zinc and iron metal cations, which are essential during collagen synthesis, flavonoids and phenolic acid esters, that are effective in reducing the inflammatory response by inhibiting the arachidonic acid lipoxygenase pathway. In addition to its significant effect on the immune system, they promote cellular phagocytic activities [29]. The caffeic acid phenethyl ester (CAPE) also has a cytoprotective function and protects against the oxidative effects of inflammatory DNA pathologies [8]. One of the discussed possible mechanisms of the antimicrobial activity of propolis is the cinnamic acid and flavonoid components, that changes the ion permeability of the inner bacterial membrane causing membrane potential dissipation and inhibition of bacterial motility [18].

One of the main characteristics attributed to propolis in the literature is its antimicrobial action [13,15,19,23,24]. The oral environment is populated by a multi-species ecosystem, some of the pathogens in the oral cavity are Streptococcus mutans, Staphylococcus aureus, Streptococcus sobrinus, and Candida albicans, which are involved in most infectious diseases of the mouth. It is well known that prevention plays an important role in caries management, therefore, anticaries activity of propolis is also demonstrated in the literature [15,23]. Asawahame et al. (2014) [23] proposed a DDS prepared using electrospinning. This DDS is biodegradable in wet environments, thus when in contact with the saliva it easily degrades. This system showed better antibacterial activity against Streptococcus mutans when compared to commercial mouthwashes and lower activity when compared to chlorhexidine. Additionally, in another study [15], the incorporation of propolis in sustained-release chitosan varnish enabled an increasing antimicrobial activity against Streptococcus mutans, Streptococcus sanguinis, Streptococcus salivarius, Lactobacillus casei, Aggregatibacter actinomycetemcomitans, Porphyromonas gingivalis, Prevotella intermedia, and Fusobacterium nucleatum compared to chlorhexidine 0.12%, chitosan-based varnish, and nystatin. In this study, sustained-release of propolis from chitosan-based varnishes showed to be a promising alternative for use in biomaterials formulations for dental caries prevention.

Another important application of propolis-based DDSs is in regenerative endodontics. The material of choice for the treatment of infectious endodontics must have an antimicrobial activity without impairing the regenerative process [8]. Studies report that propolis extract as an intracanal medication was more effective against Enterococcus faecalis compared to a mixture of tri-antibiotics [41,44], and that it showed greater antimicrobial activity associated with calcium hydroxide [34,44]. In a study that evaluated the use of propolis in endodontic therapy, the action was equally effective compared to the triple antibiotic, 2% chlorhexidine gel, and calcium hydroxide with propylene glycol against Candida albicans after 7 days at both depths into the dentinal tubules [14].

The lasting antimicrobial effect of propolis is justified by its low solubility, this is an important aspect if the biomaterial is introduced into an area of low vascularization where systemically administered antibiotics can scarcely work [8]. Studies [20,34] have demonstrated that ethanolic extract of propolis was continuously being released after 7 days. In another similar study, release profile studies demonstrated that propolis can be released from systems for an extended period (over 20 days) [45]. The heterogeneity of the results on drug release becomes a limitation of the study. These results are due not only to the origin of propolis but also to the lack of standardization of methodologies and biomaterials (DDSs) tested, according to the purpose of the application. However, there is a clear consensus that propolis can be used for long periods in DDSs.

In the study by Balata et al. (2018) [29], biodegradable chitosan chips loaded with Saudi propolis extract were developed as a controlled delivery system for pulpotomy. In this study, propolis lead to a total or partial reduction in inflammation, absence of necrosis, and greater formation of hard tissue compared to the use of formocresol. This result is consistent with other studies [46,47] that report propolis induced complete hard tissue barrier formation in pulpotomies. This can be elucidated by the anti-inflammatory activity of propolis, which promotes collagen synthesis by dentine pulp cells and stimulates the production of transforming growth factor (TGF)-1 as well as by the free radical and superoxide neutralizing components released by propolis [29].

The literature also reported the application of propolis-based DDSs in periodontics. Periodontal disease is a chronic infection resulting from a tissue response to a complex biofilm, and affects the supporting structures of the teeth (periodontium) [18,21]. One of the treatments for this condition is the systemic administration of medications and local mouthwash solutions. However, in this therapy high concentrations are used for prolonged periods, posing the risk of side effects and the emergence of resistant strains [20,22,36]. This condition also arouses interest in dentistry in the development of DDSs. Propolis becomes an excellent option due to its prolonged release in these systems and, especially, its antimicrobial activity. De Souza Ferreira et al. (2013) [24] investigated mixed propolis and metronidazole microparticles, which demonstrated in vitro antimicrobial activity against all tested strains, namely E. faecalis ATCC 51299, E. faecalis ATCC 29212, S. pyogenes ATCC 19615, S. mutans ATCC 25175, S. aureus ATCC 25923, K. pneumoniae ATCC 700608, and E. coli ATCC 25922. These microparticles have the advantages of low cost and a variety of dosage forms that can be incorporated as semi-solid systems, and administered in periodontal pockets more easily and safely. In one study [18], biodegradable polymeric PLLA/PCL films with propolis were developed for the application of guided tissue engineering and showed antibacterial activity against Staphylococcus aureus. The porosity of the substrate is essential to promote an environment of cell proliferation, the formation of new tissue, and improve vascular invasion [48]. The incorporation of propolis modified the surface topography of the films, increasing the porosity, which may be beneficial for adhesion [18]. It was also demonstrated that the addition of propolis increased the surface area associated with a fiber morphology arrangement, allowing the encapsulation and fixation of cells, which also allows prolonged release of propolis in periods longer than 48 h, making it a promising material in the engineering of mineralized dental tissues [8,11].

Due to its anti-inflammatory property, propolis can also be a support therapy in cases of oral lesions which inflicts pain and discomfort, such as recurrent aphtous stomatitis (RAS), denture stomatitis (DS), and other ulcerative conditions [35,39,40,49]. RAS has an unknown aetiology and is symptom-based, it presents as a painful rounded ulcer surrounded by an erythematous halo, while DS is a chronic disorder that affects denture-bearing patients and is associated with fungal infection (Candida albicans) [35,38,39,40,49]. Besides promoting antimicrobial activity to fight candida infection, the anti-inflammatory activity of propolis has been shown to reduce outbreak frequency, reduce ulcer size, promote a faster healing and pain relief, and therefore improve quality of life in those patients. A muco-adhesive film was prepared with propolis extract and applied to the lesion site and patients reported a longer lasting pain relief and higher overall satisfaction with the treatment, compared to placebo [38]. A 500 mg propolis or placebo capsule was administered to RAS patients for six months. Patients who received propolis daily presented a reduction in outbreak frequency and improvement in quality of life [37].

Among the engineering of mineralized dental tissues, the prolonged release of propolis over a month indicated that it could inhibit these dental pathogens in implants long-term, according to Son et al. (2021) [19]. One of the compounds responsible for the antimicrobial action of propolis, cinnamic acid derivatives, showed good stability in orally disintegrating films over twelve weeks, thus, proving to be an ideal substance for release in the oral mucosa and to control infections [25]. The use of natural actives in nanofibers has been validated for the manufacture of adhesives for oral mucosa abrasions and to fight inflammation. Propolis reduced the size of the fibers and, when released, activated hydroxyl groups present in the oral mucosa that tend to form deprotonated species at alkaline pH, providing negative charges with the ability to increase drug solubility and bioaccessibility [11].

According to the main characteristics needed to succeed in DDSs, nanosized particles are advantageous due to their size and are therefore easier to penetrate and overcome barriers at the cellular level. To provide a more efficient pharmacological therapy of oral pathologies, they can also provide bioadhesive properties that respond to stimuli through intelligent systems, as long as the particles are biocompatible and biodegradable [32]. Incorporation of new drug delivery technologies for natural products actives reduces drug degradation, minimizes side effects from cytotoxic products in non-target regions, and facilitates administration in pediatric and geriatric patients [7].

It is expected that shortly, the use of controlled delivery systems for the treatment of odontogenic and non-odontogenic diseases, in particular the use of nanoparticulate formulations, will become routine in clinical practice. It is irrefutable that there are some complexities involved in translating laboratory-developed biomaterials to industry. For this to occur, more methodologies evaluating these materials are needed as well as more government efforts to make the legislation more efficient in approving biomaterials aiming to amplify the development and commercialization of advanced drug delivery platforms.

## 5. Conclusions

It can be concluded that there is a beneficial impact on the incorporation of propolis in drug delivery systems. Although there is evidence of antimicrobial, anti-inflammatory, and regenerative activities in preclinical studies, more in-depth studies including the toxicity of this substance, a detailed physicochemical characterization, and genotoxicity assessment of biomaterials containing propolis as DDSs are necessary to ensure safe use in humans. Moreover, clinical studies must be performed to confirm the effectiveness of propolis-containing delivery systems. Overall, the authors envisage that this systematic review can aid and orientate further studies concerning the use of propolis in dental applications.

## Figures and Tables

**Table 1:** Terms used in the search strategy.

**Electronic Database** Search and Terms
**PubMed (MEDLINE)** #1 Propolis [MeSH] OR Bee Glue OR Glue, Bee OR Bee Bread OR Bread, Bee #2 Tissue Scaffolds [MeSH] OR Scaffold, Tissue OR Scaffolds, Tissue OR Tissue Scaffold OR Tissue Scaffolding OR Scaffolding, Tissue OR Scaffoldings, Tissue OR Tissue Scaffoldings OR Scaffold OR Drug Delivery Systems [MeSH] OR Delivery System, Drug OR Delivery Systems, Drug OR Drug Delivery System OR System, Drug Delivery OR Systems, Drug Delivery OR Drug Targeting OR Drug Targetings OR Targeting, Drug OR Targetings, Drug OR Nanofibers [MeSH] OR Nanofiber OR Nanospheres [MeSH] OR Nanosphere OR Hydrogels [MeSH] OR Hydrogel OR Injectable #3 Guided Tissue Regeneration [MeSH] OR Tissue Regeneration, Guided OR Regeneration, Guided Tissue OR Dentistry OR Dental OR Regenerative Dentistry OR Dental, Regenerative OR Periodontics [MeSH] OR Periodontic OR Periodontal Medicine OR Medicine, Periodontal OR Medicines, Periodontal OR Periodontal Medicines OR Periodontal OR Periodontal Regeneration OR Periodontal Engineering OR Oral Bone Regeneration OR Periapical Tissue OR Regenerative Endodontics [MeSH] OR Endodontic, Regenerative OR Endodontics, Regenerative OR Regenerative Endodontic OR Aphthous Stomatitis OR Mucosal Lesions #1 AND #2 AND #3
**Scopus** #1 ALL(“Propolis” OR “Bee Glue” OR “Glue, Bee” OR “Bee Bread” OR “Bread, Bee”) #2 ALL(“Tissue Scaffolds” OR “Scaffold, Tissue” OR “Scaffolds, Tissue” OR “Tissue Scaffold” OR “Tissue Scaffolding” OR “Scaffolding, Tissue” OR “Scaffoldings, Tissue” OR “Tissue Scaffoldings” OR “Scaffold” OR “Drug Delivery Systems” OR “Delivery System, Drug” OR “Delivery Systems, Drug” OR “Drug Delivery System” OR “System, Drug Delivery” OR “Systems, Drug Delivery” OR “Drug Targeting” OR “Drug Targetings” OR “Targeting, Drug” OR “Targetings, Drug” OR “Nanofibers” OR “Nanofiber” OR “Nanospheres” OR “Nanosphere” OR “Hydrogels” OR “Hydrogel” OR “Injectable”) #3 ALL(“Guided Tissue Regeneration” OR “Tissue Regeneration, Guided” OR “Regeneration, Guided Tissue” OR “Dentistry” OR “Dental” OR “Regenerative Dentistry” OR “Dental, Regenerative” OR “Periodontics” OR “Periodontic” OR “Periodontal Medicine” OR “Medicine, Periodontal” OR “Medicines, Periodontal” OR “Periodontal Medicines” OR “Periodontal” OR “Periodontal Regeneration” OR “Periodontal Engineering” OR “Oral Bone Regeneration” OR “Periapical Tissue” OR “Regenerative Endodontics” OR “Endodontic, Regenerative” OR “Endodontics, Regenerative” OR “Regenerative Endodontic” OR “Aphthous Stomatitis” OR “Mucosal Lesions”) #1 AND #2 AND #3
**Embase** #1 ‘Propolis’ OR ‘Bee Glue’ OR ‘Glue, Bee’ OR ‘Bee Bread’ OR ‘Bread, Bee’ #2 ‘Tissue Scaffolds’ OR ‘Scaffold, Tissue’ OR ‘Scaffolds, Tissue’ OR ‘Tissue Scaffold’ OR ‘Tissue Scaffolding’ OR ‘Scaffolding, Tissue’ OR ‘Scaffoldings, Tissue’ OR ‘Tissue Scaffoldings’ OR ‘Scaffold’ OR ‘Drug Delivery Systems’ OR ‘Delivery System, Drug’ OR ‘Delivery Systems, Drug’ OR ‘Drug Delivery System’ OR ‘System, Drug Delivery’ OR ‘Systems, Drug Delivery’ OR ‘Drug Targeting’ OR ‘Drug Targetings’ OR ‘Targeting, Drug’ OR ‘Targetings, Drug’ OR ‘Nanofibers’ OR ‘Nanofiber’ OR ‘Nanospheres’ OR ‘Nanosphere’ OR ‘Hydrogels’ OR ‘Hydrogel’ OR ‘Injectable’ #3 ‘Guided Tissue Regeneration’ OR ‘Tissue Regeneration, Guided’ OR ‘Regeneration, Guided Tissue’ OR ‘Dentistry’ OR ‘Dental’ OR ‘Regenerative Dentistry’ OR ‘Dental, Regenerative’ OR ‘Periodontics’ OR ‘Periodontic’ OR ‘Periodontal Medicine’ OR ‘Medicine, Periodontal’ OR ‘Medicines, Periodontal’ OR ‘Periodontal Medicines’ OR ‘Periodontal’ OR ‘Periodontal Regeneration’ OR ‘Periodontal Engineering’ OR ‘Oral Bone Regeneration’ OR ‘Periapical Tissue’ OR ‘Regenerative Endodontics’ OR ‘Endodontic, Regenerative’ OR ‘Endodontics, Regenerative’ OR ‘Regenerative Endodontic’ OR ‘Aphthous Stomatitis’ OR ‘Mucosal Lesions’ #1 AND #2 AND #3
**Web of Science** TS = (Propolis OR Bee Glue OR Glue, Bee OR Bee Bread OR Bread, Bee) TS = (Tissue Scaffolds OR Scaffold, Tissue OR Scaffolds, Tissue OR Tissue Scaffold OR Tissue Scaffolding OR Scaffolding, Tissue OR Scaffoldings, Tissue OR Tissue Scaffoldings OR Scaffold OR Drug Delivery Systems OR Delivery System, Drug OR Delivery Systems, Drug OR Drug Delivery System OR System, Drug Delivery OR Systems, Drug Delivery OR Drug Targeting OR Drug Targetings OR Targeting, Drug OR Targetings, Drug OR Nanofibers OR Nanofiber OR Nanospheres OR Nanosphere OR Hydrogels OR Hydrogel OR Injectable) TS = (Guided Tissue Regeneration OR Tissue Regeneration, Guided OR Regeneration, Guided Tissue OR Dentistry OR Dental OR Regenerative Dentistry OR Dental, Regenerative OR Periodontics OR Periodontic OR Periodontal Medicine OR Medicine, Periodontal OR Medicines, Periodontal OR Periodontal Medicines OR Periodontal OR Periodontal Regeneration OR Periodontal Engineering OR Oral Bone Regeneration OR Periapical Tissue OR Regenerative Endodontics OR Endodontic, Regenerative OR Endodontics, Regenerative OR Regenerative Endodontic OR Aphthous Stomatitis OR Mucosal Lesions) #1 AND #2 AND #3
**Cochrane** #1 Propolis OR Bee Glue OR Glue, Bee OR Bee Bread OR Bread, Bee #2 Tissue Scaffolds OR Scaffold, Tissue OR Scaffolds, Tissue OR Tissue Scaffold OR Tissue Scaffolding OR Scaffolding, Tissue OR Scaffoldings, Tissue OR Tissue Scaffoldings OR Scaffold OR Drug Delivery Systems OR Delivery System, Drug OR Delivery Systems, Drug OR Drug Delivery System OR System, Drug Delivery OR Systems, Drug Delivery OR Drug Targeting OR Drug Targetings OR Targeting, Drug OR Targetings, Drug OR Nanofibers OR Nanofiber OR Nanospheres OR Nanosphere OR Hydrogels OR Hydrogel OR Injectable #3 Guided Tissue Regeneration OR Tissue Regeneration, Guided OR Regeneration, Guided Tissue OR Dentistry OR Dental OR Regenerative Dentistry OR Dental, Regenerative OR Periodontics OR Periodontic OR Periodontal Medicine OR Medicine, Periodontal OR Medicines, Periodontal OR Periodontal Medicines OR Periodontal OR Periodontal Regeneration OR Periodontal Engineering OR Oral Bone Regeneration OR Periapical Tissue OR Regenerative Endodontics OR Endodontic, Regenerative OR Endodontics, Regenerative OR Regenerative Endodontic OR Aphthous Stomatitis OR Mucosal Lesions #1 AND #2 AND #3

**Table 2:** Demographic data and main results of studies.

Study	Country of Study	Study Design	Objective	Application	Biomaterial	Type of Propolis	Propolis Origin	Characterization of Propolis	Drug Release	Toxicity	Controls	Simple Size	Main Findings
[8]	Romania	In vitro study	Designed a composite biomaterial based on a high viscosity soft propolis extract (70% propolis) and shell clam, with antiseptic and osteoinductive qualifies for hard tissue regeneration.	Mineralized tissue engineering that can be used in dentistry	Bioactive cement with antiseptic and osteoinductive qualities	Aqueous extract 70%	Commercial 70% soft propolis extract, Bioremed, Romania.	Scanning electronic microscopy (SEM), reversed-phase liquid chromatography (HPLC) with diode array detection, proliferation assay, and differentiation assay	Not available	Cell viability and adhesion level with human dental papilla cell line.	The cells cultured in normal culture condition (DMEM-F12 (Sigma-Aldrich) supplemented with 10% FCS (Hyclone), 1% Antibiotic-antimycotic (Sigma-Aldrich)	n = 3	The shell-propolis-based biomaterial promoted and sustained human stem cells attachment, proliferation, and differentiation, presenting an important osteoinductive effect essential for the mineralized tissue reparation process.
[9]	Brazil	In vitro study	Development and characterization of semisolid systems containing propolis or sildenafil prepared from Carbopol 934P and poloxamer 407	Endodontics in pulp protection	Binary polymeric systems containing poloxamer 407 (P407) and Carbopol 934P (C934P) were designed to deliver propolis extract (PE)	Ethanolic extract	Propolis was collected from an experimental apiary in the farm of the State University of Maringa (Parana State, Brazil).	Rheological analysis by ViscoStar− Plus R controlled shear rate rotating viscometer	50% in 500 min	Not available	Sildenafil citrate was purchased from Pfizer (Dongcheng District, Beijing, China)	n = 3 for all tests, except bioadhesive strength (n = 5)	The data obtained in these formulations indicated a potentially useful role in pulp protection, however, clinical evaluation is necessary.
[11]	Italy	In vitro study	The optimization of the electrospinning process to fabricate zein electrospun nanofibres (ZN) loaded with propolis (PZN).	Tissue inflammation in the presence of oral lesions	Electrospun fibers	Hydroalcoholic solution	Informed only the location Erbaflor, Italy	Not available	70% in 24 h	Not available	Pure propolis	n = 20	The zein nanofibers can guarantee a sustained release of propolis directly to the target, providing a more efficient solution for treatments based on the administration of ‘one shot’ of the active ingredient, minimizing side effects.
[14]	Malaysia	In vitro study	The antifungal activity of propolis, triple antibiotic paste (TAP), 2% chlorhexidine gel, and calcium hydroxide with propylene glycol was evaluated on root canal dentinal tubules.	Candida albicans-infected root canal dentinal tubules	Intracanal medicaments	Ethanolic extract	Stakich, Royal Oak, Michigan, USA	Not available	Not available	Not available	Triple antibiotic paste (TAP), 2% chlorhexidine gel and calcium hydroxide with propylene glycol	n = 18	Propolis demonstrated comparable efficacy to triple antibiotic paste, 2% chlorhexidine gel, and calcium hydroxide with propylene glycol in inhibiting the growth of C. albicans at both depths over a period of 7 days.
[15]	Brazil	In vitro study	Antimicrobial activity of sustained-release propolis-based chitosan varnish useful on dental cariogenic biofilm prevention	Anti-cariogenic agent	Propolis—based chitosan varnish	Ethanolic extract	Green propolis was collected from commercial beekeeping named Pharmanéctar^®^ in Minas Gerais State, Brazil.	Provided by fabricant PharmaNectar, Brazil, 2007, not described the techniques was used	20% in 24 h	Not available	Chlorhexidine 0.12%, chitosan-based varnish and nystatin	n = 5	Sustained-release chitosan-based propolis varnishes (5%, 10%, and 15%) inhibited all tested microorganisms, deserving clinical studies to confirm it is in vivo activity.
[18]	Turkey	In vitro study	Produce barrier membranes from biodegradable polymers, namely, PLLA and PCL, with an antibacterial feature promoted by propolis.	Guided tissue regeneration in periodontology	Biodegradable polymer films	Ethyl alcoholic extract	Not informed	Not available	Not available	Not available	Antibiotic disk including 30 μgr chloramphenicol (C30) (HIMEDIA)	n = 5	Propolis has a positive influence on the thermal, mechanical, and degradation properties of the blend films to achieve the required values for GTR. Also, films with propolis showed antibacterial activity against Gram (+) bacteria.
[19]	Korea	In vitro study	The potential of propolis-embedded zeolite nanocomposites for dental implant application.	Dental implants	Propolis-embedded zeolite nanocomposites	Aqueous extract	Propolis extracts were purchased from Rapha Propolis Co., (Jeonju, Korea).	Fourier transform-infrared spectra (FT-IR)	90% in 30 days	MTT cell cytotoxicity assay	PLA/PCL pellets containing propolis-embedded zeolite nanocomposites	Not informed	Eluted propolis solution from PLA/PCL pellets showed significant antibacterial efficacy against *C. albicans*, *S. mutans*, and *S. sobrinus*.
[20]	Brazil	In vitro study	Development and characterization of semisolid systems containing propolis prepared from carbomer 934P and poloxamer 407 (P407)	Periodontal pocket for the treatment of periodontitis	Semisolid Systems	Ethanolic extract	Propolis was collected from an experimental apiary in the farm of the State University of Maringa (Parana State, Brazil)	Not available	80% in 168 h	Not available	Formulations without propolis microparticles	n = 5	The release profile studies showed that propolis could be released from the systems for an extended period (more than 7 days). The properties of the candidate formulations indicate a potential advantageous role in the treatment of periodontal diseases.
[21]	Malaysia	In vitro study	Formulated periodontal chips from Malaysian propolis in chitosan base and to evaluate the physical, biological and antibacterial properties.	Treatment of chronic periodontitis	Biodegradable periodontal chips	Ethanolic extract	Raw propolis purchased from Ayer Keroh, Malacca, Malaysia	Not available	80% in 6 days	Not available	Chlorhexidine (0.2%, *w*/*v*) and ethanol (20%, *w*/*v*)	n = 15 for all tests, except surface morphology and thickness (n = 35)	Malaysian propolis can be evaluated into a chip and be used in treating patients with periodontal disease. It was found to be biodegradable have a high release rate, and have antimicrobial activity against gram-positive and gram-negative bacteria.
[22]	Brazil	In vitro study	Development of a novel liquid crystalline system containing MNPs and propolis	Periodontal pockets	A liquid crystalline system containing iron oxide magnetic nanoparticles (MNPs)	Ethanolic extract	Propolis was acquired from the Iguatemi Experimental Farm of the State University of Maringa, Parana state, Brazil.	Folin-Ciocalteu method	36% in 120 h	Cytotoxicity by micro-crustacean *Artemia salina* and fibroblasts cell line.	A system without propolis	n = 6	The system containing propolis and magnetic nanoparticles displays important in vitro fungicide activity, which was increased when an alternating external magnetic field was applied, indicating a potential alternative therapy for the treatment of periodontal disease.
[23]	Thailand	In vitro study	Antibacterial activity against Streptococcus mutans and the inhibition of adhesion on a smooth glass surface during the biofilm formation was tested.	Mouth-dissolving dosage form and as an anti-cariogenic agent	Propolis-PVP electrospun fibers	Ethanolic extract 5% (*w*/*v*)	Propolis was obtained from Chiangmai Healthy Product Co., Ltd. (Chiangmai, Thailand).	Not available	Not available	Not available	Chlorhexidine mouthwash solution (0.12%) 1 mg/mL	n = 8 (SEM); n = 3 (antimicrobial assays)	The results indicated the potential of electrospun fibers to be used as mouth-dissolving fibers for effective antibacterial activity in the oral cavity.
[24]	Brazil	In vitro study	The antimicrobial activity of microparticles was evaluated against some microorganisms of periodontal importance.	Periodontal pockets	Ethylcellulose microparticles	Ethanolic extract	Three samples of propolis from Apis mellifera L. beehives were collected at apiaries in the Northeast of Paraná state, Brazil.	Determination of total flavonoid content and, determination of total phenol content by Folin-Ciocalteu method	20% in 32 h	Not available	Metronidazole	n = 3 for all tests, except the determination of total flavonoid content and total phenol content (n = 6)	The strains of Enterococcus faecalis, Streptococcus pyogenes, and Streptococcus mutans were more susceptible to the propolis and E. faecalis to the metronidazole.
[25]	Brazil	In vitro study	Production and characterization of orally disintegrating films from gelatin and hydrolyzed collagen containing the ethanol extract of propolis.	Control oral infection	Films of gelatin and hydrolyzed collagen	Ethanolic extract	12-type resin (Star Rigel Raf- ^~^ fard, Sao Paulo, Brazil)	Folin–Ciocalteau method, Fourier transform infrared spectroscopy (FTIR) and, scanning electron microscopy	80% in 15 min	Not available	Films without the ethanol extract of propolis	n = 10 for all tests, except SEM (n = 16); in vitro release and antimicrobial assay (n = 3)	The ethanol extract of propolis produced the antimicrobial activity in the film as well as provided a better resistance matrix and increased mucoadhesiveness.
[29]	Egypt	In vitro study and animal model	The formulation of commercial Saudi propolis into biodegradable chitosan chips and evaluation of its effectiveness as a pulpotomy agent.	Treatment of vital pulpotomy	Saudi Propolis into biodegradable chitosan chips	Ethanolic extract	Propolis (El Akbr)^®^ was obtained from a honey bee market located in Jeddah, Saudi Arabia (El Maher shop, Wadi El Nahil Co., Taeif, Saudi Arabia).	Determination of total phenolic content, determination of total flavonoid content, determination of the antioxidant activity of the extract, quantification of polyphenolic constituents	35% in 7 days	Histopathological evaluation	Formocresol	n = 6 (in vitro); n = 18 (animal model)	Formulation of propolis extract as chitosan biodegradable chips can be used effectively for local sustained propolis delivery into the infected periodontal pockets, as it results in the production of higher quality secondary dentin with the less inflammatory response of the pulp.
[34]	Pakistan	Randomized clinical trial	Assessing the effect of Chinese propolis paste as an intracanal medicament on postoperative endodontic pain intensity	Endodontics treat in necrotic teeth with periapical radiolucency	Paste	Propolis powder	FMBP. Henan Fumei Biotechnology Co., Ltd., Changge, China, reg no.: 411082100010933	Not available	Not available	Not available	Calcium hydroxide 20%.	n = 40	The effect of propolis was found to be comparable to the calcium hydroxide group in managing postoperative endodontic pain, with no reported adverse effects.
[35]	India	Randomized clinical trial	Assess the effectiveness of applying topical propolis for the treatment of oral lichen planus.	Oral lichen planus:	Topical propolis	Not informed	Not informed	Not available	Not available	Not available	Triamcinolone acetonide 0.1%	n = 27	Topical propolis demonstrated comparable effectiveness to triamcinolone acetonide 0.1% in managing oral lichen planus (OLP).
[36]	Iran	In vitro study	A mucoadhesive gel formulation incorporating a concentrated extract of propolis was developed for the treatment of periodontitis.	Periodontitis	Mucoadhesive gel	Propolis particles	Agricultural Research Center (Isfahan, Iran)	Folin–Ciocalteu method for determination of polyphenol contents. Aluminum chloride colorimetric method was used to determine flavonoid content	80% em 7 days	Not available	Tetracycline disc (30 µg/mL)	n = 3	Drug release assay demonstrated that propolis exhibited a prolonged release from the system, lasting more than 7 days. Additionally, propolis exhibited a substantial growth inhibition zone against *Porphyromonas gingivalis*.
[37]	USA	Randomized clinical trial	Daily ingestion of one 500-mg capsule of propolis will reduce the frequency of outbreaks of recurrent aphthous stomatitis	Aphthous ulcers	500-mg capsule of propolis	Incapsuled	Vitamin World	Not available	Not available	Not available	Placebo capsule of a calcium-based food supplement	n = 10 (propolis group) n = 9 (placebo group)	Daily ingestion of 500 mg of propolis can potentially reduce the frequency of aphthous ulcer episodes, particularly those who have not found relief through alternative treatment methods.
[38]	Egypt	Clinical and in vitro studies	Treating aphthous ulceration by maintaining a therapeutic level of the active ingredient in the mouth for a prolonged period of time and enhancing drug absorption	Aphthous ulcers	Niosomal oromuco-adhesive films	Commercial propolis	Imtenan Health Co., Egypt	The content of total flavonoid compounds was determined by an aluminum chloride colorimetric assay. The content of total phenolic compounds was determined by the Folin-Ciocalteau assay	64% in 8 h	Not available	Placebo group	n = 3 (in vitro); n = 24 (clinical study)	In the group receiving medication, the reduction in ulcer size was observed as early as the second and third day of treatment. Complete healing was achieved within the first 10 days, and the pain relief lasted for more than 4–5 h, which was in stark contrast to the placebo group.
[39]	Iran	Randomized clinical trial	Assess the potential impact of this product in reducing the occurrence of recurrent aphthous ulcers.	Aphthous ulcers	500-mg capsule of propolis	Incapsuled	Not informed	Not available	Not available	Not available	Placebo group	n = 22	Propolis group exhibited a lower number of relapses compared to the placebo group. Moreover, significant reductions in the number and size of lesions, pain levels, and recovery time were observed in the propolis group.
[40]	United Arab Emirates	Clinical and in vitro studies	Formulations of buccal pastes containing propolis were developed and subjected to both pharmaceutical and clinical evaluations for the treatment of recurrent aphthous stomatitis.	Aphthous ulcers	Buccal paste	Ethanolic extract	Hajj Seed local farms (Dubai, UAE)	Not available	Not available	Not available	Control formula (placebo)	n = 3 (in vitro); n = 120 (clinical study)	The healing rate of aphthous ulcers was significantly higher compared to the placebo group. Furthermore, the size of the ulcers decreased within the first day of application. Patients in the propolis groups experienced a significant reduction in pain intensity within the initial 24 h, with 90% of patients reporting relief, compared to only 35% in the placebo group.

**Table 3:** Risk of bias in in vitro studies.

	D1. Bias in Planning and Allocation			D2. Bias in Sample/Specimen Preparation		D3. Bias in Outcome Assessment		D4. Bias in Data Treatment and Outcome Reporting	
Authors, Year	1.1 Control Group	1.2 Randomizatio n of Samples	1.3 Sample Size Rationale and Reporting	2.1 Standardizatio n of Samples and Materials	2.2 Identical Experimental Conditions across Groups	3.1 Adequate and Standardized Testing Procedures and Outcomes	3.2 Blinding of the Testing Operator	4.1 Statistical Analysis	4.2 Reporting Study Outcomes
[8] Simu et al., 2018	Sufficiently Reported	Not reported	Not reported	Insufficiently reported	Insufficiently reported	Sufficiently reported	Not reported	Insufficiently reported	Insufficiently reported
[9] Fabri et al., 2011	Sufficiently Reported	Not reported	Not reported	Insufficiently Reported	Sufficiently reported	Sufficiently reported	Not reported	Sufficiently reported	Sufficiently reported
[11] Bonadies et al., 2019	Sufficiently reported	Not reported	Not reported	Sufficiently reported	Sufficiently reported	Sufficiently reported	Not reported	Not reported	Insufficiently reported
[14] Chua et al., 2014	Sufficiently Reported	Not reported	Not reported	Sufficiently Reported	Sufficiently reported	Insufficiently reported	Not reported	Sufficiently reported	Sufficiently reported
[15] Franca et al., 2014	Sufficiently Reported	Not reported	Not reported	Sufficiently Reported	Sufficiently reported	Sufficiently reported	Not reported	Insufficiently reported	Insufficiently reported
[18] Ahi et al., 2019	Sufficiently Reported	Not reported	Not reported	Sufficiently reported	Sufficiently reported	Sufficiently reported	Not reported	Not reported	Insufficiently reported
[19] Son et al., 2021	Sufficiently reported	Not reported	Not reported	Sufficiently reported	Insufficiently reported	Sufficiently reported	Not reported	Not reported	Insufficiently reported
[20] Bruschi et al., 2013	Sufficiently Reported	Not reported	Not reported	Sufficiently Reported	Sufficiently reported	Sufficiently reported	Not reported	Sufficiently reported	Sufficiently reported
[21] Al-Bayaty et al., 2017	Sufficiently Reported	Not reported	Not reported	Sufficiently Reported	Insufficiently reported	Sufficiently reported	Not reported	Insufficiently reported	Insufficiently reported
[22] de Alcântara Sica de Toledo et al., 2018	Sufficiently Reported	Not reported	Not reported	Sufficiently reported	Sufficiently reported	Sufficiently reported	Not reported	Sufficiently reported	Sufficiently reported
[23] Asawahame et al., 2014	Sufficiently Reported	Not reported	Not reported	Insufficiently Reported	Sufficiently reported	Insufficiently reported	Not reported	Not reported	Insufficiently reported
[24] de Souza Ferreira et al., 2013	Sufficiently Reported	Not reported	Not reported	Sufficiently Reported	Insufficiently reported	Insufficiently reported	Not reported	Not reported	Insufficiently reported
[25] Borges et al., 2015	Sufficiently Reported	Not reported	Not reported	Insufficiently Reported	Sufficiently reported	Sufficiently reported	Not reported	Not reported	Insufficiently reported
[29] Balata et al., 2018	Sufficiently Reported	Not reported	Not reported	Sufficiently Reported	Sufficiently reported	Insufficiently reported	Not reported	Not reported	Insufficiently reported
[37] Arafa et al., 2018	Not reported	Not reported	Not reported	Inufficiently reported	Sufficiently reported	Sufficiently reported	Not reported	Sufficiently reported	Sufficiently reported
[39] Ali and Abdul Rasool, 2011	Sufficiently reported	Not reported	Not reported	Sufficiently reported	Sufficiently reported	Sufficiently reported	Not reported	Insufficiently reported	Insufficiently reported
[41] Aslani and Malekpour, 2016	Sufficiently reported	Not reported	Not reported	Sufficiently reported	Sufficiently reported	Sufficiently reported	Not reported	Not reported	Insufficiently reported

**Table 4:** Risk of bias in non-randomized clinical studies.

Author, Year	1.1 Bias due to Counfounding	1.2 Bias in Selection of Participants into the Study	2.1 Bias in Classification of Interventions	3.1 Bias due to Deviations from Intended Interventions	3.2 Bias due to Missing Data	3.3 Bias in Measurement of Outcomes	3.4 Bias in Selection of the Reported Result
[39] Ali and Rassol, 2011	Low	Low	Low	Low	Low	Low	Low

**Table 5:** Risk of bias in randomized clinical trials.

Author, Year	1. Randomization Process	2. Deviations from Intended Interventions	3. Missing Outcome Data	4. Measurement of the Outcome	5. Selection of the Reported Result	Overall Risk of Bias
[34] Shabbir et al., 2020	Low	Some concerns	Some concerns	Low	Low	Some concerns
[35] Zenouz et al., 2015	Low	Some concerns	Some concerns	Low	Low	Some concerns
[36] Samet et al., 2005	Some concerns	Some concerns	Some concerns	Low	Low	Some concerns
[37] Arafa et al., 2018	Low	Some concerns	Some concerns	Low	Low	Some concerns
[38] Delavarian et al., 2020	Some concerns	Some concerns	Some concerns	Low	Low	Some concerns

## Data Availability

The data presented in this study are openly available in Open Science Framework at https://doi.org/10.17605/OSF.IO/V9FXT.
